# Auditory brainstem function in women with vestibular migraine: a controlled study

**DOI:** 10.1186/s12883-019-1368-5

**Published:** 2019-06-27

**Authors:** Alice A. Takeuti, Mariana L. Fávero, Erica Helena Zaia, Fernando F. Ganança

**Affiliations:** 1 Departamento de Otorrinolaringologia e Cirurgia de Cabeça e Pescoço, Universidade Federal de São Paulo, São Paulo, Brazil; 2 Divisão de Educação e Reabilitação dos Distúrbios da Comunicação (DERDIC), Pontíficia Universidade Catolica de São Paulo, São Paulo, Brazil; 3 Audio-Vestibular Clinic, Vancouver, Canada

**Keywords:** Migraine, Vertigo, Auditory evoked potentials, Brainstem, Hyperacusis

## Abstract

**Background:**

Vestibular migraine (VM) has been recognized as a diagnostic entity over the past three decades. It affects up to 1% of the general population and 7% of patients seen in dizziness clinics. It is still underdiagnosed; consequently, it is important to conduct clinical studies that address diagnostic indicators of VM. The aim of this study was to assess auditory brainstem function in women with vestibular migraine using electrophysiological testing, contralateral acoustic reflex and loudness discomfort level.

**Methods:**

The study group consisted of 29 women with vestibular migraine in the interictal period, and the control group comprised 25 healthy women. Auditory brainstem response, frequency following response, binaural interaction component and assessment of contralateral efferent suppression were performed. The threshold of loudness discomfort and the contralateral acoustic reflex were also investigated. The results were compared between the groups.

**Results:**

There was a statistically significant difference between the groups in the frequency following response and the loudness discomfort level.

**Conclusions:**

The current study suggested that temporal auditory processing and loudness discomfort levels are altered in VM patients during the interictal period, indicating that these measures may be useful as diagnostic criteria.

## Background

The link between vestibular symptoms and migraine has led to the publication of diagnostic criteria for a new disorder called vestibular migraine [1–3]. Although there is a high frequency of auditory symptoms associated with migraine, there is inadequate information regarding how migraine occurs; vestibular assessments performed via hearing tests do not provide enough specific data. Psychoacoustic evaluations are normal in most cases despite reports of hearing loss in cases of acute migraine and vestibular migraine; this hearing loss is most likely as a result of cochlear vasospasms [3–8].

Specific auditory symptoms such as phonophobia (vestibular migraine and migraine without aura diagnosis criteria) and hearing loss and tinnitus (brainstem migraine with aura diagnosis criteria) suggest impairment of auditory pathways in migraine cases [2].

Auditory brainstem responses (afferent and efferent, electric conduction, binaural interaction, temporal processing and evoked potential suppression by efferent auditory system) can be assessed with electrophysiological exams such as auditory brainstem response, binaural interaction component, frequency following response and assessments of efferent pathways by suppression of auditory brainstem responses.

Brainstem electric disorders can also affect the stapedius muscle reflex or the acoustic reflex due to dysfunctions of higher auditory centers or supratentorial structures [9, 10].

The aim of this study is to assess auditory brainstem function in women with vestibular migraine by means of electrophysiological testing and acoustic reflex thresholds and to verify the presence of hyperacusis according to loudness discomfort level, as defined by Nields et al. [11]. The hypothesis of this study is that women with vestibular migraine have altered auditory brainstem function due to the relationship between the trigeminovascular system and central and peripheral auditory structures.

## Methods

### Patients

This case-control study enrolled 29 women with vestibular migraine (diagnosed according to the criteria of Neuhauser et al., modified by the Bárány Society and International Headache Society [2] and by the 3rd edition of The International Classification of Headache Disorders [12]) in the study group during the interictal period of vestibular migraine. The control group comprised 25 healthy women matched to the study group by age. The subjects in the study group were recruited from the vestibular migraine outpatient clinic of a neurotology service and represent all patients who fulfilled the inclusion criteria during the collection period. The control group subjects were volunteers.

All participants were screened for normal hearing (i.e., ≤ 25 dB HL at octave frequencies from 250 to 8000 Hz) and normal middle ear function (i.e., no history of chronic ear disease, normal otoscopy and normal 226 Hz tympanometry). To avoid any unintentional bias regarding the interictal period, a new interview was conducted to confirm the absence of current symptoms before tests.

The exclusion criteria, for both groups, were acute or chronic neurological, neurotological (other than vestibular migraine in the study group), or psychiatric disorders and/or the use of migraine prophylactic drugs, central nervous system-active drugs or contraceptives.

The procedures used in this study were approved by the ethical committee (no. 43437015.2.0000.5505). All subjects in this study agreed to and signed the informed consent form.

### Evoked potentials acquisition

Electrophysiologic tests were conducted using the Intelligent Hearing System (Smart EP windows USB version 3.91) with insert earphones ER-3A. Responses were collected with silver chloride electrodes and were differentially recorded from Cz (active) to the ipsilateral mastoid (reference), with a common grounding electrode placed on the forehead. The subjects were instructed to lie comfortably in the supine position and relax to promote a passive recording condition. No other stimulus was used during the tests.

### Frequency following response recording

The frequency following response was evoked by 1024 sweeps and 60 dBHL tone bursts at 500 Hz using rarefaction polarity, with a 5000 μs rise and fall time in a trapezoidal envelope and a 15,000-μs duration at a rate of 5.1 per sec. The response was filtered between 30 and 3000 Hz in a graduated analysis window of 50 msec. The response cycles obtained for the study group were compared to those obtained for the control group.

Auditory brainstem response recording.

The auditory brainstem response was evoked by 1024 clicks with a 100-μs duration using a rarefaction polarity and a rate of 37.1 clicks per sec. A high-pass filter of 100 Hz and a low-pass filter of 3000 Hz were used on a 12.5-msec graduated window of analysis. Waveforms were obtained in both ears at 70 dBHL, and waves I, III and V were identified. The absolute latencies of waves I, III and V and interpeak latencies I-III, III-V and I-V were obtained and compared between the study group and control group.

### Auditory brainstem response suppression recording

To assess the suppression of the auditory brainstem response by the efferent pathway, a 60-dBHL intensity white noise was used in the contralateral ear (Matas et al., 2010). Waveforms were obtained at 70 dBHL in the following order:

- Right ear response without contralateral noise;
- Right ear response with contralateral noise;
- Left ear response without contralateral noise;
- Left ear response with contralateral noise;

The absolute latencies for waves I, III and V and the interpeak I-III, III-V and I-V latencies without noise were subtracted from the results with contralateral noise, and the results were compared between the groups. The right and left ear results for the study group individuals were compared with the right and left ear results for the control group individuals, respectively.

### Binaural interaction component recording

The binaural interaction component test was evoked by 1024 sweeps of 100 μs clicks with rarefaction polarity at 70 dBHL and a rate of 37.1 stimuli per sec. A high-pass filter of 100 Hz and a low-pass filter of 3000 Hz were used for simultaneous unilateral and bilateral recording within a graduated analysis window of 12.5 msec. Three recordings were obtained:

- Monoaural right ear stimulation and right ear potential recording;
- Monoaural left ear stimulation and left ear potential recording;
- Binaural stimulation and right and left ear potential recording.

The binaural interaction component trace was obtained by the sum of the monoaural recording (1 and 2) followed by the subtraction of the binaural recording [3]: (right ear + left ear) – binaural. These analyses were performed using a tool in the IHS system. The latency and amplitude of the binaural interaction component waveform were compared between the study group and the control group.

### Loudness discomfort level assessment

Loudness discomfort levels were obtained for pure tones between 0.25 and 8 kHz in both ears using an AC33 Interacoustics® audiometer. The initial presentation level was 50 dBHL, and each stimulus was presented for approximately 2 s, with 1-s intervals between presentations. The presentation level was increased gradually by 5 dBHL until a discomfort sensation was evoked. The participants were instructed to raise their hands when they experienced minimal discomfort, at which point the presentations were interrupted [13].

The loudness discomfort level values were used to determine the hyperacusis level: negative (discomfort at 95 dBHL or more at all frequencies), mild (discomfort level between 80 and 90 dBHL at two or more frequencies), moderate (discomfort level between 65 and 75 dBHL at two or more frequencies), and severe (discomfort level at 60 dBHL or less at two or more frequencies) [14].

### Contralateral acoustic reflex assessment

Contralateral acoustic reflex testing was performed at 500, 1000, 2000 and 4000 Hz for each ear in both groups using AT235 Interacoustics® equipment, with pure tone presentations lasting 1.5 s. Each stimulus was presented for 1.5 s at an initial intensity of 70 dBHL and was increased by 5-dBHL steps until the acoustic reflex was obtained. The reflex was considered absent when no response was obtained at 110 dBHL. Recruitment was defined as a difference of less than 75 dBHL between the acoustic reflex threshold and the hearing threshold at each frequency.

### Data analysis

The main researcher used Microsoft Excel 2011® was used to record and store the data. A descriptive analysis of the data that considered absolute and relative frequencies, central tendency measures (average and median) and dispersion measures (standard deviation, minimum and maximum values) was performed.

For quantitative variables, the standard distribution was verified, and Student’s t-test was used to compare the groups. The equality of variance (standard deviation square) was not assumed when the homogeneity of a certain variable could not be confirmed. For the association analyses between independent qualitative variables and outcome measures, the chi-square test was used. For statistical significance, a descriptive level of 5% (*p* < 0.05) was considered. Data were entered in Excel and analyzed with the Statistical Package for the Social Sciences (SPSS), version 22.0 for Windows.

## Results

In this study, 54 women were analyzed (25 in the control group and 29 in the study group). Their ages ranged from 23 to 74 years, with an average of 49.7 years (SD = 13.1) and a median of 49. There was no statistically significant difference in age between the groups (*p* = 0.573).

There was no statistically significant difference between the average latency and amplitude of binaural interaction component values when the study group and the control group were compared. The same finding was observed for the absolute and interpeak latencies obtained for the auditory brainstem response and auditory brainstem response suppression (*p* > 0.05).

The frequency following response latencies of the study group had average values that were significantly higher than those of the control group in both ears (*p* < 0.05), except for the latency of wave I in the left ear (*p* = 0.102). The frequency following response interpeak I-III variable in the left ear differed significantly between the groups (*p* = 0.003). The average for the study group was 2.00 msec (SD = 4.1), while the average for the control group was 1.6 msec (SD = 0.52) (Table 1).

**Table 1:** Frequency Following Response latencies in the control group and study group

	*CG*			*SG*			
	n	mean	SD	n	mean	SD	p*
*FFR Right ear*							
Latency I	25	4.91	0.40	29	5.40	0.76	*0.004* ^*¥*^
Latency II	25	6.58	0.62	29	7.31	0.91	*0.001*
Latency III	25	8.40	0.88	29	9.25	1.10	*0.003*
Latency IV	25	10.29	1.06	29	11.06	1.31	*0.023*
Latency V	25	12.21	1.25	29	13.00	1.40	*0.035*
Latency VI	25	14.08	1.28	29	15.10	1.61	*0.014*
Interpick I-II	25	1.68	0.49	29	1.91	0.43	0.067
Interpick II-III	25	1.82	0.51	29	1.94	0.44	0.325
Interpick III-IV	25	1.89	0.38	29	1.81	0.38	0.413
Interpick IV-V	25	1.92	0.42	29	1.94	0.39	0.823
Interpick V-VI	25	1.87	0.48	29	2.10	0.60	0.134
*FFR Left ear*							
Latency I	25	5.05	0.44	29	5.36	0.87	0.102^¥^
Latency II	25	6.66	0.70	29	7.36	0.99	*0.004*
Latency III	25	8.32	0.94	29	9.20	1.11	*0.003*
Latency IV	25	10.18	1.03	29	11.19	1.41	*0.005*
Latency V	25	12.10	1.18	29	13.04	1.46	*0.013*
Latency VI	25	14.00	1.36	29	15.11	1.73	*0.012*
Interpick I-II	25	1.60	0.52	29	2.00	0.41	*0.003*
Interpick II-III	25	1.67	0.51	29	1.84	0.41	0.165
Interpick III-IV	25	1.86	0.36	29	1.99	0.59	0.357
Interpick IV-V	25	1.91	0.41	29	1.85	0.36	0.545
Interpick V-VI	25	1.90	0.43	29	2.07	0.45	0.158

* *t-Student test;* ^¥^ equality of variance not assumed

*FFR* Frequency Following Response*, CG* Control group, *SG* Study group

There was a statistically significant difference in the average loudness discomfort level threshold of the right ear between the groups at 250 Hz (*p* = 0.006), 500 Hz (*p* = 0.013) and 3000 Hz (*p* = 0.023). When analyzing the left ear, significant differences between groups were noted for the frequencies of 500 Hz (*p* = 0.02), 1000 Hz (*p* = 0.014), 2000 Hz (p = 0.01) and 3000 Hz (p = 0.02), with higher levels obtained for the control group (Table 2).

**Table 2:** Loudness Discomfort Level means in the study and control groups

	*CG*			*SG*			
	n	mean	SD	n	mean	SD	p*
250 Hz *RE*	16	104.06	7.12	16	95.94	8.41	*0.006*
500 Hz *RE*	20	107.50	9.67	18	98.33	11.88	*0.013*
1000 Hz *RE*	21	105.00	8.06	19	99.47	12.01	0.103^¥^
2000 Hz *RE*	18	103.06	9.87	18	96.11	11.19	0.056
3000 Hz *RE*	19	104.21	10.04	19	95.53	12.46	*0.023*
4000 Hz *RE*	16	103.44	12.87	18	95.00	12.72	0.064
6000 Hz *RE*	10	99.50	12.79	15	95.33	12.32	0.423
8000 Hz *RE*	7	89.29	11.34	11	85.45	7.57	0.400
250 Hz *LE*	13	98.46	8.99	16	92.81	10.48	0.136
500 Hz *LE*	21	106.43	11.08	19	97.63	11.83	*0.020*
1000 Hz *LE*	22	105.68	10.04	20	97.00	11.74	*0.014*
2000 Hz *LE*	20	105.25	11.41	18	95.28	11.18	*0.010*
3000 Hz *LE*	20	104.50	11.23	18	95.56	11.49	*0.020*
4000 Hz *LE*	15	101.67	11.90	18	96.39	13.15	0.240
6000 Hz *LE*	12	104.17	13.11	15	95.33	13.69	0.102
8000 Hz *LE*	7	87.86	12.54	10	85.50	11.41	0.693

*t-Student test; ^¥^ equality of variance not assumed

*CG* Control group, *SG* Study group, *RE* Right ear, *LE* Left ear

When analyzing the loudness discomfort level classification, statistically significant differences were observed for both the right and left ears. For the right ear, women with mild hyperacusis tended to be more likely to be in the study group than in the control group (52% versus 18%; *p* = 0.019). This tendency was verified in the left ear (*p* = 0.039) (Table 3).

**Table 3:** Classification of the Loudness Discomfort Level in the study group and control group

	*CG*		*SG*			Total	
	n	(%)	n	(%)	p	n	(%)
*LDL RE*							
Negative level^a^	18	(81)	10	(47)	*0.019*	28	(65)
Mild level^b^	4	(18)	11	(52)		15	(34)
*LDL LE*							
Negative level^a^	18	(81)	9	(45)	*0.039*	27	(64)
Mild level^b^	4	(18)	10	(50)		14	(33)
Moderate level^c^	0	(0)	1	(5)		1	(2)
Total	22	(100)	21	(100)		43	(100)

^a^Discomfort level at 95 dBHL or more; ^b^discomfort level between 80 and 90 dBHL in two or more frequencies; ^c^discomfort level between 65 and 75 dBHL in two or more frequencies

*LDL* Loudness Discomfort Level*, CG* Control group, *SG* Study group, *RE* Right ear, *LE* Left ear

For the acoustic reflex threshold, there was no significant difference between the study group and the control group (*p* > 0.05).

## Discussion

In the current research, patients with vestibular migraine presented increased latencies of the frequency following response and lower discomfort thresholds compared to the control group, and the differences were statistically significant. These findings suggest altered pure tone temporal processing and mild level hyperacusis [11], respectively.

These results suggest that the trigeminovascular system has an important influence on auditory brainstem function, even in the interictal period of vestibular migraine, when the data were collected; this finding indicates that the mechanism of vestibular migraine involves permanent alterations in subcortical auditory pathways.

The involvement of the brainstem and the diencephalon in migraines has been described [15], as has the involvement of the trigeminovascular system [16]. The relationship between these structures and the central and peripheral auditory systems has also been described [17, 18].

The frequency following response latencies were significantly longer in the patients with vestibular migraine than in the control group, suggesting altered pure tone temporal processing, which may also affect the processing of complex sounds. The topographic register of the frequency following response is the representation of the acoustic signal in cephalic regions of the brainstem, such as the inferior colliculus and lateral lemniscus [19–21]. These findings complement those of Agessi et al. [22], who reported worse outcomes for temporal resolution during central auditory processing testing in migraine patients. The lack of similar studies of the frequency following response in vestibular migraine and migraine limits the literature comparison.

The lower discomfort thresholds suggest the presence of mild hyperacusis, in concordance with other previous studies. Sand and Vingen [23] and Sand et al. [24] hypothesized that this sound sensitivity could be related to subcortical disinhibition at the level of the activation nuclei, such as the inferior colliculus in the brainstem and the medial geniculate nucleus in the thalamus, reinforcing the hypothesis that migraine is triggered by a bottom-up mechanism, as other authors have also described [15, 25–28]. The relationship between low levels of serotonin in migraine and subcortical disinhibition, including that of the pontomesencephalic auditory pathways, in the inner ear and vestibular nuclei, has been demonstrated in previous studies [23, 28–30].

The remaining electrophysiologic tests did not show any significant difference, suggesting that binaural hearing, efferent auditory brainstem response suppression and auditory brainstem response are not altered in patients with vestibular migraine during the interictal period.

To date, there are no studies on the binaural interaction component in vestibular migraine or migraine. According to Shore et al. [18], the medial superior olivary complex, the main binaural interaction component structure, does not have any connection with the trigeminal ganglion, despite its anatomical proximity to structures supplied by the trigeminal nerve, such as the cochlear nucleus, lateral superior olivary complex and trapezoidal body. Additionally, Agessi et al. [22] did not find any disorder in the dichotic digits test of central auditory processing in patients with migraine; this test assesses figure-ground ability, which is based on binaural hearing. In concordance with these authors, the present study suggests that there are no abnormalities of binaural integration in patients with vestibular migraine.

Auditory brainstem response suppression with contralateral white noise in vestibular migraine did not yield any significant differences between the study group and the control group. This finding contrasts with other studies that used transient otoacoustic emission to evaluate contralateral suppression [6, 31]. A possible reason is that the two tests involve different anatomical structures and analyze different responses: the outer hair cells and their otoacoustic emission are assessed in the contralateral suppression of transient otoacoustic emission [32], while the auditory nerve and auditory brainstem pathways and their electrical responses are assessed with efferent auditory brainstem response suppression.

As previous studies have suggested [33–35], there were no differences in auditory brainstem click responses between groups during the migraine interictal period. However, other authors found significant differences [36, 37]. These conflicting results could be related to the duration of the illness and the frequencies of migraine crisis [38]. The parameters used during the registration of auditory evoked potentials could also be related.

The contralateral acoustic reflex threshold analysis did not show a statistically significant difference between the two groups, most likely due to the small role of the tensor timpani muscle vs the stapedius muscle in triggering the acoustic reflex. The interneurons involved in tensor timpani muscle contraction receive descending impulses from the inferior colliculus, the superior olivary complex, serotoninergic sources and high cerebral centers. Although these areas are also involved in migraine and vestibular migraine, the participation of tensor timpani muscle contraction may not be strong enough to modify the acoustic reflex thresholds. The stapedius muscle does not have a direct anatomical relationship with vestibular migraine [39].

The auditory temporal processing brainstem disorder evidenced by the frequency following response and the mild hyperacusis observed in this study reinforce the hypothesis that the inferior colliculus has an important role in migraine and vestibular migraine pathophysiology. The inferior colliculus is part of the extralemniscal auditory pathway, a multisensorial system that is likely involved in the auditory symptoms of migraine. The neurons of the inferior colliculus dorsal nucleus connect to the auditory thalamocortical system. Connections between the inferior and the superior colliculus influence saccadic eye movements and other important motor responses to acoustic stimuli, which are essential for sound localization. The extralemniscal auditory system thus responds to both auditory and non-auditory stimuli, such as somatosensory, visual and vestibular stimuli. Auditory symptoms such as tinnitus, hyperacusis and discriminatory alterations could be related to the abnormal activation of this pathway [40].

The results of this study complement the knowledge that sensory input processing is abnormal in vestibular migraine and migraine [23, 27] and involves brainstem dysfunction. It also reinforces the established role of a bottom-up mechanism in the physiology of vestibular migraine [29]. More studies on the extension of migraine spectra are necessary to clarify the physiopathology and determine the involvement of all other sensory modalities, thus allowing the development of new treatment strategies.

Some limitations of this study are as follows: 1) the relatively small number of patients; 2) the cross-sectional design, which limits a longitudinal overview of the test results; and 3) the unknown duration of illness.

## Conclusions

The current study suggested that temporal auditory processing and the loudness discomfort level are altered in vestibular migraine patients during the interictal period and may be used as diagnostic criteria.

## Data Availability

The datasets used and analyzed during the current study are available from the corresponding author on reasonable request.

## Abbreviations

VM: Vestibular migraine

## References

[1] Neuhauser H, Leopold M, von Brevern M, Arnold G, Lempert T (2001). The interrelations of migraine, vertigo and migrainous vertigo. Neurology.

[2] Lempert T, Olesen J, Furman J, Waterston J, Seemungal B, Carey J, Bisdorff A, Versino M, Evers S, Newman-Toker D (2012). Vestibular migraine: diagnostic criteria - consensus document of the Bárány society and the international headache society. J Vestib Res.

[3] Furman JM, Marcus DA, Balaban CD (2013). Vestibular migraine: clinical aspects and pathophysiology. Lancet Neurol.

[4] Lee H, Whitman GT, Lim JG, Yi SD, Cho YW, Ying S, Baloh RW (2003). Hearing symptoms in migrainous infarction. Arch Neurol.

[5] Battista RA (2004). Audiometric findings of patient with migraine-associated dizziness. Otol Neurootol.

[6] Bolay H, Bayazit YA, Gündüz B, Ugur AK, Akcali D, Altunyay S, Ilica S, Babacan A (2008). Sub-clinical dysfunction of cochlea and cohlear efferents in migraine: an otoacoustic study. Cephalalgia..

[7] Neff B, Staab J, Eggers S, Carlson M, Schmitt W, Van Abel K, Worthington D, Beatty C, Driscoll C, Shepard N (2012). Auditory and vestibular symptoms and chronic subjective dizziness in patients with Meniere’s disease, vestibular migraine and Meniere’s disease with concomitant vestibular migraine. Otol Neurotol.

[8] Lempert T (2013). Vestibular Migraine. Semin Neurol.

[9] Campos MI, Reis CJ (1989). Reflexo do músculo do estapédio alterado em portadores de epilepsia com audição normal. Rev bras otorrinolaringol.

[10] Carmel P, Star A (1963). Acoustic and non acoustic factors modifying midlle ear muscle activity in waking cats. J Neuro-Oncol.

[11] Nields JA, Fallon BA, Jastreboff PJ (1999). Carbamazepine in the treatment of Lyme disease–induced Hyperacusis. J Neuropsychiatry Clin Neurosci.

[12] Headache Classification Committee of the International Headache Society (IHS) (2018). The International Classification of Headache Disorders, 3rd edition. Cephalalgia..

[13] Knobel KAB, Sanchez TG (2006). Nível de desconforto para sensação de intensidade em indivíduos com audição normal. Pró-Fono R Atual Cient.

[14] Goldstein B, Shulman A (1996). Tinnitus - Hyperacusis and the loudness discomfort level test - a preliminary report. Int Tinnitus J.

[15] Akerman S, Holland PR, Goadsby PJ (2011). Diencephalic and brainstem mechanisms in migraine. Nat Rev Neurosci [Internet].

[16] Stankewitz A, Aderjan D, Eippert F, May A (2011). Trigeminal nociceptive transmission in migraneurs predicts migraine attacks. J Neurosci.

[17] Vass Z, Shore SE, Nuttall a L, Miller JM (1998). Direct evidence of trigeminal innervation of the cochlear blood vessels. Neuroscience..

[18] Shore S, Vass Z, Wys N, Altschuler R (2000). Trigeminal ganglion innervates the auditory brainstem. J Comp Neurol.

[19] Moushegian G, Rupert AL, Stillman RD (1973). Scalp-recorded early responses in man to frequencies in the speech range. Electroencephalogr Clin Neurophysiol.

[20] Du Y, Kong L, Wang Q, Wu X, Li L (2011). Auditory frequency-following response: a neurophysiological measure for studying the “cocktail-party problem”. Neurosci Biobehav Rev.

[21] Gockel HE, Krugliak A, Plack CJ, Carlyon RP (2015). Specificity of the human frequency following response for carrier and modulation frequency assessed using adaptation. J Assoc Res Otolaryngol.

[22] Agessi LM, Villa TR, Dias KZ, Carvalho D, Pereira LD (2014). Central auditory processing and migraine: a controlled study. J Headache Pain [Internet].

[23] Sand T, Vingen JV (2000). Visual, long-latency auditory and brainstem auditory evoked potentials in migraine: relation to pattern size, stimulus intensity, sound and light discomfort thresholds and pre-attack state. Cephalalgia..

[24] Sand T, Zhitniy N, White LR, Stovner LJ (2008). Brainstem auditory-evoked potential habituation and intensity-dependence related to serotonin metabolism in migraine: a longitudinal study. Clin Neurophysiol.

[25] Lance JW, Lambert GA, Goadsby PJ, Duckworth JW (1983). Brainstem influences on the cephalic circulation: experimental data from cat and monkey of relevance to the mechanism of migraine. Headache: The Journal of Head and Face Pain.

[26] Weiller C, May A, Limmroth VA, Jüptner M, Kaube H, Kaube H, Schayck RV, Coenen HH, Diener HC (1995). Brainstem activation in spontaneous human migraine attacks. Nat Med.

[27] Murdin L. Audiovestibular sensory processing in migraine. PhD [Thesis migrania] London. University College London, 2010.

[28] Bhargava VK, McKean CM (1977). Role of 5-hydroxytryptamine in the modulation of acoustic brainstem (far-field) potentials. Neuropharmacology..

[29] Hamel E (2007). Serotonin and migraine: biology and clinical implications. Cephalalgia..

[30] Marano E, Marcelli V, Di Stasio E, Bonuso S, Vacca G, Manganelli F (2005). Trigeminal stimulation elicits a peripheral vestibular imbalance in migraine patients. Headache..

[31] Joffily L (2016). de Melo Tavares de Lima MA, Vincent MB, Frota SMMC. Assessment of otoacoustic emission suppression in women with migraine and phonophobia. Neurol Sci.

[32] Favero ML, Sanchez TG, Bento RF, Nascimento A (2006). Contralateral suppression of otoacoustic emission in patients with tinnitus. Braz J Otorhinolaryngol.

[33] Dash AK, Panda N, Khandelwal G, Lal V, Mann SS (2008). Migraine and audiovestibular dysfunction: is there a correlation?. Am J Otol Head Neck Med Surg.

[34] Firat Y, Ozturan O, Bicak U, Yakinci C, Akarcay M (2006). Auditory brainstem response in pediatric migraine: during the attack and asymptomatic period. Int J Pediatr Otorhinolaryngol.

[35] Arakaki X, Galbraith G, Pikov V, Fonteh AN, Harrington MG (2014). Altered brainstem auditory evoked potentials in a rat central sensitization model are similar to those in migraine. Brain Res.

[36] Boyer Nelly, Dallel Radhouane, Artola Alain, Monconduit Lénaïc (2014). General trigeminospinal central sensitization and impaired descending pain inhibitory controls contribute to migraine progression. Pain.

[37] Hamed SA, Youssef AH, Elattar AM (2012). Assessment of cochlear and auditory pathways in patients with migraine. Am J Otolaryngol.

[38] Tessitore A., Russo A., Esposito F., Giordano A., Taglialatela G., De Micco R., Cirillo M., Conte F., d’Onofrio F., Cirillo S., Tedeschi Gioacchino (2011). Interictal cortical reorganization in episodic migraine without aura: an event-related fMRI study during parametric trigeminal nociceptive stimulation. Neurological Sciences.

[39] Mukerji S, Windsor AM, Lee DJ (2010). Auditory brainstem circuits that mediate the middle ear muscle reflex. Trends Amplif.

[40] Moller AR (2006). Hearing: Anatomy, Phisiology and Disorders of the Auditory System. 2nd ed.

